# Biochemical biomarkers of skin mucus in *Neogobius melanostomus* for assessing lead pollution in the Gulf of Gorgan (Iran)

**DOI:** 10.1016/j.toxrep.2019.12.003

**Published:** 2020-01-03

**Authors:** Fakhriyeh Omidi, Hojatollah Jafaryan, Rahman Patimar, Mohammad Harsij, Hamed Paknejad

**Affiliations:** a Department of Fisheris, Faculty of of Natural Resources, Department of Fisheries, University of Gonbad Kavous, Gonbad Kavous, Iran; b Department of Fisheris, Faculty of of Natural Resources, Department of Fisheries, Gorgan University of Agricultural Sciences and Natural Resources, Gorgan, Iran

**Keywords:** Gulf of Gorgan, Lead contamination, Mucosal biomarker, *Neogobius melanostomus*

## Abstract

The present study was conducted to provide basic information on the physiological damages (mucosal indicators) of Round Goby under laboratory and environmental conditions of lead contamination. The other goal was to find mucosal biomarkers of lead contamination in the Gulf of Gorgan. In the laboratory study, 400 pieces of fish with an average the weight of 35 ± 7.16 g were caught alive and were exposed to lead concentrations of 0, 3.75, 7.5 and 15 mg / l for 14 days. Once the experiment was completed, the samples of mucus and liver were collected from the fish samples with the samples being surveyed by analyses of SDS-PAGE, mucus safety and toxicology. In the field test, the samples of fish, water, and sediment were prepared from four stations. In addition to measuring the lead in water, sediment and liver tissue, the samples of mucus from the caught fish were similarly analyzed under the laboratory conditions mentioned. According to the results under laboratory conditions, obvious differences were observed between the protein pattern of samples of treatment with the control group. Further, among innate immune parameters of the mucus, only lysozyme showed a significant correlation with the concentrations of lead accumulated in the liver of Round Goby (P < 0.05). Under environmental conditions, obvious differences were also observed between the protein pattern of samples of contaminated stations with the control station. Further, among innate immune parameters of the mucus, lysozyme indicated a positive correlation while alkaline phosphatase showed a negative correlation with lead contamination in the liver of Round Goby (P < 0.05). In total, in the pattern of mucus protein, the intensity of the band within the range of 25 kDa was enhanced in both conditions, suggesting the presence of proteases in this region. Also, among the immune parameters, lysozyme enzyme showed a significant and positive correlation with liver lead contamination in both conditions (P < 0.05). These can be considered as the biomarkers of protein pattern and safety in Round Goby mucus for assessing lead pollution in Gulf of Gorgan.

## Highlights

• Skin mucus is a key component of the innate immune system against metals pollution.
• Lead produced a clear change in the protein profile of mucus in Round Goby.
• The amount of biochemical parameters of mucus changed upon exposure to lead.
• The enhancement of band intensity at 25 kDa was proposed as a biomarker.
• The elevation of lysozyme level was proposed as a biomarker.

## 1 Introduction

Metal pollution is a serious issue because of its potential accumulation in both the environment and living creatures [1]. Heavy metals can enter an aquatic ecosystem from numerous natural and anthropogenic sources, including domestic or industrial sewage, leaching from landfills, severe wind runoff, shipping and harbor operations, and atmospheric deposits [2,3]. Lead (Pb) is an extremely toxic metal in aquatic environments [4]. Pb exposure can be deadly for aquatic animals even at low levels due to bioaccumulation [5], which causes an extensive range of toxic effects on physiological functions in aquatic animals [6].

The bioaccumulation of metals in tissues of aquatic organisms has been previously reported [[7], [8], [9]]. In order to protect aquatic organisms, it is necessary to determine contamination levels of trace elements through chemical biomonitoring and evaluation of biomarkers that represent early indicators of biological effects [7].

Biomarkers trace the secondary effects of pollutants on aquatic organisms. They survey aquatic physiological status for assessing the aquatic health and finally aquatic ecosystems [10]. The use of biochemical parameters of mucus as an indicator is gaining popularity for studying the effects of sub-lethal stresses on fish. The skin of fish is covered by a mucus layer, which is continuously changed. It acts as a physical obstacle between the fish and aquatic environment. Notably, mucus has evolved a variety of innate immune factors, including immunoglobulins, proteases, lysozyme, lectins, proteolytic enzymes and diversity of other antibacterial proteins and peptides [11]. The composition and extent of secretion of epidermal mucus have been reported in response to microbial, physical, and environmental stressors such as maturity stage, density, season, acidity, and salinity [12,13]. Meanwhile, little information has been previously reported over the effect of heavy metals on the composition and amount of secretion of fish skin mucus [[14], [15], [16]].

Studies have shown that fish are a good option for studying the effects of heavy metals in aquatic ecosystems. The reason is that they are at higher levels of the food pyramid, they have more biological and ecological values, and information on fish is more available than other aquatic animals [17]. The family Gobiidae is one of the most diverse fish families in the world; they are generally benthic and live near the beach most times of the year [18]. They feed on small crustaceans, mollusks, annelid worms, small fishes, and eggs of various invertebrates [19]. As they are not utilized and are abundant species, and because of their extensive population in the Caspian Sea, they play an important role in the general production of this sea.

The Gulf of Gorgan is a semiclosed gulf, which is located at the southeast end of the Caspian Sea. The length of this gulf is 33 km, its width is 12 km, and its maximum depth is 4 m. This gulf is separated from the Caspian Sea by the Miankaleh Peninsula [20], with many small rivers entering it.

The growth of the human population and development of agricultural as well as industrial activities have led to an increase in various pollutants, most of which find their way to the Gulf of Gorgan. It is expected that the production of these pollutants will increase dramatically in the upcoming years. Therefore, this study investigated the determination of mucosal biomarkers of lead contamination in the Gulf of Gorgan using ecophysiological indicators (protein pattern and mucus immunological components) of Round Goby as a biological model.

## 2 Materials and methods

### 2.1 Study region

**Fig. 1:**
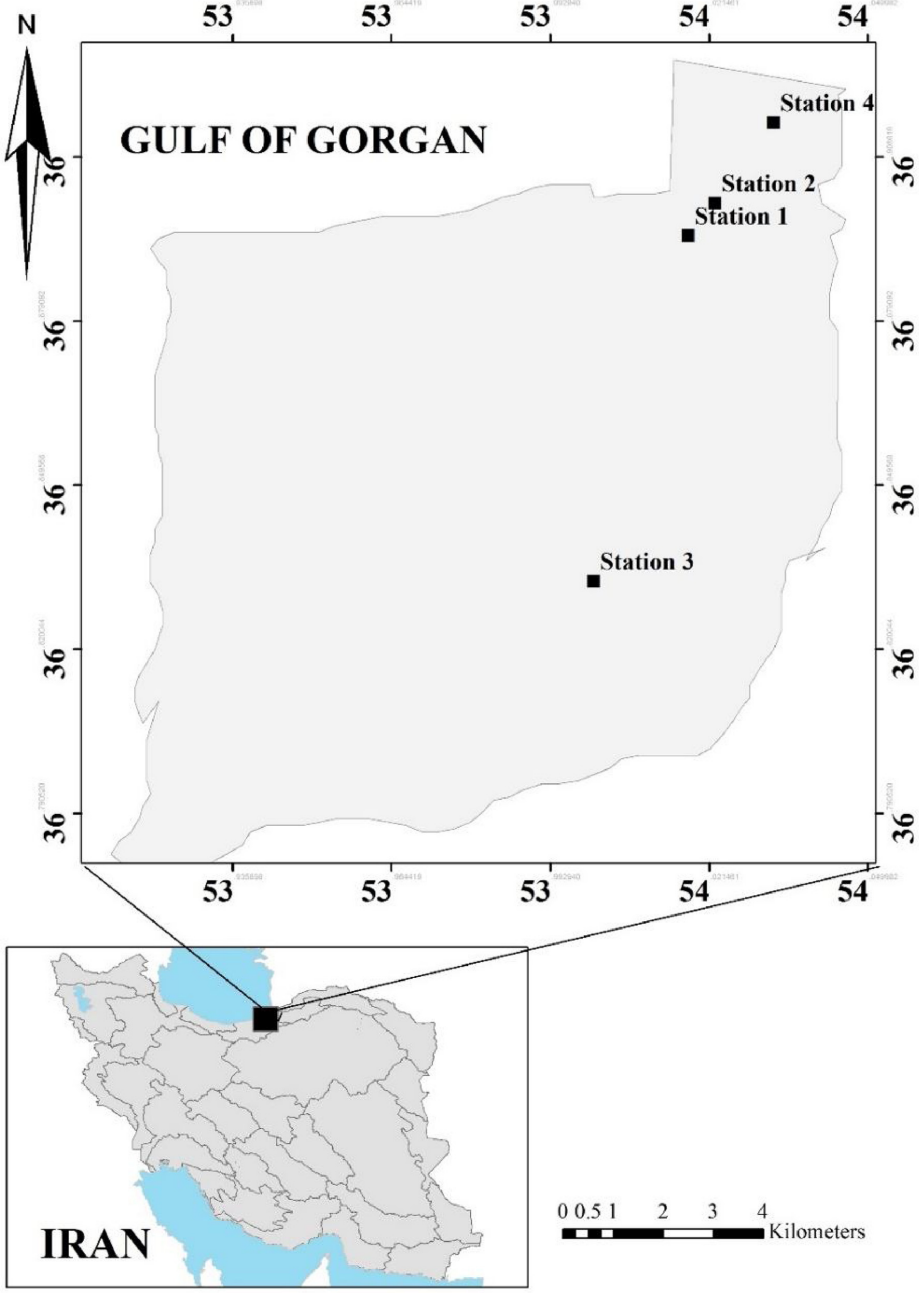
Map of selected stations for the environmental study.

This research was conducted in the summer of 2018 in the Gulf of Gorgan, Iran (Fig. 1). The studied region was divided into several sampling stations according to the accessibility of the region, as well as fishing and input pollution load. Stations 1, 2 and 3 are located in the more inner parts of the gulf than station 4 which is located in the vicinity of the inlet mouth of the gulf. They are categorized as less polluted (stations 1 & 2) and polluted (stations 3 & 4). Here, station 1 was considered as control, and the geographical coordinates of the sampling stations were as follows: 36° 49′ to 36° 54′ N and 54° 1′ to 54° 2′ E.

### 2.2 The laboratory conditions

#### 2.2.1 Experimental fish collection and maintenance

A total of 400 pieces of *Neogobius melanostomus* with an average weight of 35 ± 7. 16 g were caught alive from station 1 (a region with less pollution) in the Gulf of Gorgan and acclimated to laboratory conditions for 14 days. For the entire duration of the experiment, the fish were maintained in glass aquariums (80-L) containing filtered sea water and equipped with oxygenation. During this period, temperature (25−27 °C), salinity (20 g/l), as well as nitrite and nitrate levels were measured and kept constant. During the acclimatization period, the fish were maintained under natural light/dark cycle and fed every day with chopped fresh fish. The water required to replace the reservoirs was transferred to the laboratory from non-contaminated parts in the Gulf of Gorgan and then was filtered.

#### 2.2.2 Determination of 96-h lethal concentration (LC50)

For this experiment, eight concentrations were considered: the nominal concentrations of 1, 10, 20, 40, 60, 80, 160 and 320 mg /l lead (II) nitrate. For each concentration, three replicates were considered, and in each glass aquarium (80-L), seven pieces of fish were randomly distributed. Also, the mortality rate was recorded daily for 4 days. Then, 96-h lethal concentration (LC50) was determined using Probit software.

#### 2.2.3 Sub-lethal exposure

The experiment was carried out for 14 days at concentrations of 0, 7.5, 15, and 30 % of LC50 lead (II) nitrate, being equal to 0, 6, 12 and 24 mg/l of lead (II) nitrate. The effective concentrations of Pb at the determined concentrations were 0, 3.75, 7.5 and 15 mg / l, respectively (molar mass of lead (II) nitrate = 331.2 g/mol, molar mass of Pb = 207.2 g/mol). Glass aquariums with a capacity of 80-L were used, with 3 replicates for each level. The fish were randomly determined and divided into aquariums (10 fish per aquaria). They were fed twice daily with chopped fresh fish (3 % of body weight) at 10:00 and 14:00 h. Note that the sub-lethal test was reproducible. The experimental water was replaced every day and the amount of lead in the water was renewed. Water quality parameters during the experiment were as follows: temperature (25−27 °C), pH (6–7.5), salinity (20 g/l), nitrite below 0.05 mg /l, and dissolved oxygen concentrations above 6 mg /l. The fish were sampled after 24 h of starvation

#### 2.2.4 Liver tissue analysis

The samples of fish were beheaded, the liver tissues were collected, and freeze-dried. The liver tissues were weighed (approx. 0.5 g dry) and then digested. The digested solutions were diluted with double-deionized water and subjected to atomic absorption spectrophotometry. The liver tissue digestion was carried out according to MOOPAM standard method described by ROPME [21]. Pb levels were measured using an atomic absorption spectrometer (Varian – Spectra, 220 FS).

#### 2.2.5 Skin mucus collection and analysis

Skin mucus collection: Mucus was collected according to the method described by Ross et al. [22], with minor modifications. Mucus was collected from five Round Goby per aquarium. The fish were transferred to zip packs containing 10 ml of 50 mM NaCl. After 1 min, fish were eliminated and released to aquariums. The mucus samples were transferred to 15 ml sterile centrifuge tubes and centrifuged (1500 *g* for 10 min at 4 °C). The obtained skin mucus was instantly frozen to prevent any external bacterial pollution, then lyophilized and stockpiled at −80 °C.

##### 2.2.5.1 Sds-Page

The protein pattern of the aqueous extract of skin mucus was surveyed by sodium dodecyl sulphate-polyacrylamide gel electrophoresis (SDS-PAGE) according to the method described by Laemmli [23], where the migration buffer consisted of 25 mM Tris and 192 mM glycine (pH 8.5). The samples were mixed with a sample buffer containing 0.01 M Triseglycine buffer (pH 6.8), 0.1 % SDS, 1 % 2-b-mercaptoethanol, and 20 % glycerol. They were then heated at 100 °C for 4 min, centrifuged 3 min at 1000 *g* (2K15 Sigma), and applied to the gel, which was run under a constant current [30 mV (7 %) and 50 mV (15 %)]. After running, the gel was stained with Coomassie Brilliant Blue G. Further, Lactalbumin (14.4 kDa), trypsin inhibitor (20.1 kDa), carbonic anhydrase (30 kDa), albumin (67 kDa), and phosphorylase b (94 kDa) were used as molecular weight markers (Pharmacia).

##### 2.2.5.2 The concentration of soluble protein

The protein concentration present in each sample was measured by the method of Lowry et al. [24], using serum albumin (BSA, Sigma), as the standard.

##### 2.2.5.3 The concentration of immunoglobulin M (IgM)

IgM was measured by the method of Siwicki and Anderson [25]. First, the protein concentration present in each sample was measured and then polyethylene glycol 12 % was added to the samples at 1:1 ratio. After incubation of the samples for 2 h at room temperature and darkness, the samples were centrifuged (5000 rpm for 5 min), and protein concentration in the upper part of the solution was measured by the method of Lowry et al. [24]. Note that polyethylene glycol causes sedimentation of the immunoglobulin M present in the protein. The immunoglobulin M content is calculated based on the difference in soluble protein concentration in the prototype and the soluble protein concentration after the addition of polyethylene glycol.

##### 2.2.5.4 Lysozyme activity

Lysozyme activity was measured using a turbidometric assay [11] and a spectrophotometer with minor modifications. Briefly, *Micrococcus lysodeikticus* was suspended in sodium acetate buffer (139 mg of sodium acetate, 17 μl of glacial acetic acid per 100 ml of water, pH 5.5). Then, 250 μl of mucus samples was well mixed with 1.250 ml of bacterial suspension, and the reduction in absorbance of the samples was recorded at 450 nm for 10 min. A unit of lysozyme activity was defined as the amount of enzyme that causes a reduction in the absorbance of 0.001 per min.

##### 2.2.5.5 Alkaline phosphatase activity

Alkaline phosphatase activity was determined using commercially available kits (Pars Azmon, Iran) and spectrophotometer (405 nm).

### 2.3 The environmental conditions

#### 2.3.1 Sampling procedure

A total of 200 pieces of fish were caught from all stations with an average weight of 28.6 ± 3.87 g. In each station, after catching the fish, mucus and liver tissue samples were prepared in three replications through the procedure described under the laboratory conditions. Then, they were transferred to the laboratory for analysis in the procedure mentioned. In addition, samples of water and sediment were also prepared in three replications from each sampling station. In the vicinity of the ice, they were transferred to the laboratory for measuring the amount of lead in water, sediment, and liver tissue using the atomic absorption spectrophotometer and the procedure described by ROPME [21].

### 2.4 Statistical analysis

Kolmogorov–Smirnov test was used to determine the normality of the data in this research. Then, the data were analyzed by one-way ANOVA and Duncan test (level of confidence 95 %). Finally, the correlation test (Pearson) was used to determine the correlation between the measured lead concentrations in the fish liver and the measured immunological indicators of mucus. The data of this study were analyzed using software R-3.5.2.

## 3 Results

### 3.1 Determination of 96-h LC50

The mean lethal concentration (LC50) values at 24, 48, 72 and 96 h of exposure were 130.50, 123.40, 89.00 and 80.00 mg/l of lead (II) nitrate, respectively (with 95 % confidence limits).

### 3.2 Lead concentrations in water, sediment, and liver samples

The concentration of lead in the water of sampling stations ranged from 0.1–3.45 μg / l (Table 1). Except for station 3, the amount of lead in water across different stations did not show a significant difference with the control station (station 1) (P > 0.05). The highest amount of lead was found at station 3. Unlike water lead, the levels of lead in the sediments of different stations had a significant difference with the control station (P < 0.05). These results were fully consistent with coastal activities in the vicinity of the areas. The concentration of lead in the sediment was 6000–12000.500 μg / kg dry weight of the sediment.

**Table 1:** Lead levels in water and sediments of sampling stations in the Gulf of Gorgan.

Stations	Lead in water (μg/l)	Lead in sediment (μg/kg)
Station 1	0.10 ± 0.00^b^	6000.00 ± 1000^c^
Station 2	0.50 ± 0.40^b^	8000.00 ± 0.00^b^
Station 3	3.45 ± 0.35^a^	12,000.500 ± 500^a^
Station 4	0.40 ± 0.10^b^	11000.500 ± 500^a^

Different letters indicate significant differences among concentrations ; ANOVA significance: p < 0.05.

The concentration of lead in liver tissues of fish at stations 1, 2, 3 and 4 was 37.44, 33.81, 60.69, and 57.86 μg / kg dry weight, respectively (Table 2). The results of lead measurement at different stations revealed a significant increase in lead concentration in the liver of fish at contaminated stations compared to the control station (station 1), (P < 0.05). The correlation coefficient between lead concentration in sediment and lead concentration in fish liver showed a large numerical value (r = 0.90, p < 0.01), while the correlation coefficient between the lead concentration in the liver of the fish and in water of its fishing area was lower (r = 0.62, p < 0.05), compared to the sediment. The levels of lead in the liver of the fish exposed to the lead concentrations under the laboratory conditions are shown in Table 2. The lead accumulation in the liver of these fish was 45.14, 45.64, 57.84, and 98.65 μg / kg dry weight, such that with the increase in the lead sub-lethal concentrations, liver lead levels increased significantly (P < 0.05).

**Table 2:** Lead levels in the liver samples of Round Goby under environmental and laboratory conditions.

Laboratory conditions		Environmental conditions	
Treatment	Concentration of liver lead (μg/kg)	Stations	Concentration of liver lead (μg/kg)
Control (0 mg/l)	45.64 ± 0.49^C^	Station 1	37.44 ± 1.00^b^
Treatment 1 (3.75 mg/l)	45.14 ± 1.00^C^	Station 2	33.81 ± 3.61^b^
Treatment 2 (7.5 mg/l)	57.84± 1.50^b^	Station 3	60.69 ± 1.31^a^
Treatment 3 (15 mg/l)	98.65 ± 2.65^a^	Station 4	57.86 ± 4.14^a^

Different letters indicate significant differences among concentrations ; ANOVA significance: p < 0.05.

### 3.3 Determination of protein pattern in lead sub-lethal concentrations

**Fig. 2:**
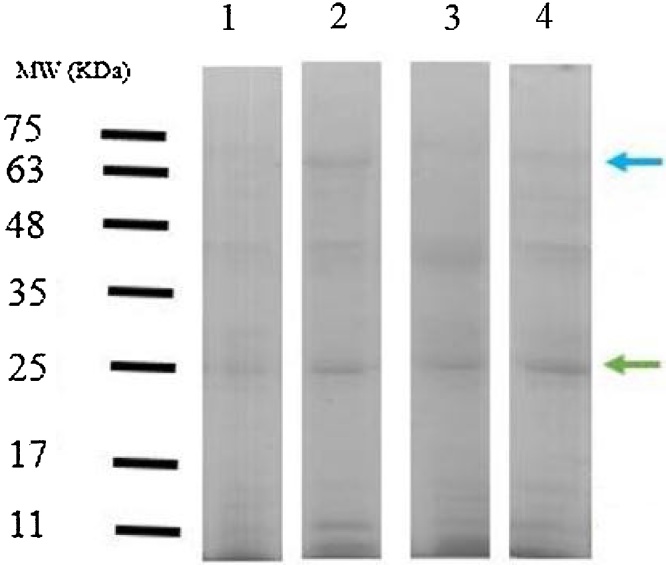
Evaluation of skin mucus protein profile using SDS-PAGE; the green arrow indicates a band with enhanced intensity; the blue arrow indicates a new band; Line 1: Control, Line 2: 3.75 mg /l, Line 3: 7.5 mg / l, Line 4: 15 mg / l lead.

**Fig. 3:**
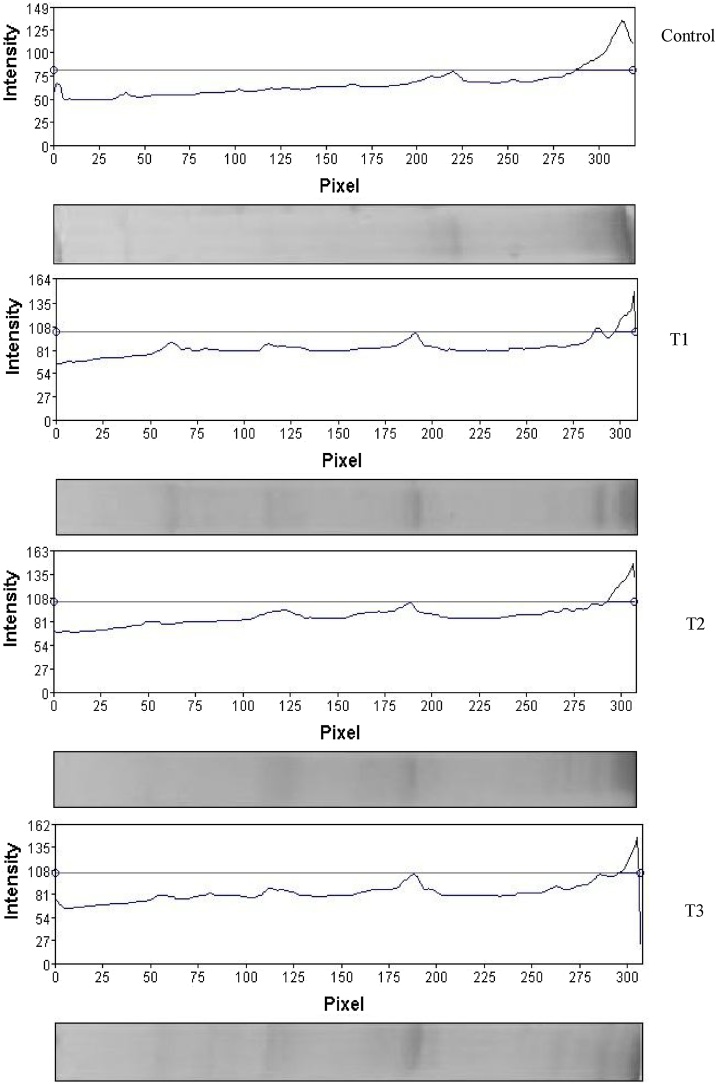
The effect of different concentrations of lead on the pattern of skin mucus protein bands under laboratory conditions.

The SDS-page of the protein pattern of the mucus in Round Goby is shown in Fig. 2, Fig. 3. Obvious differences were observed between the protein pattern of treated and control groups. Protein pattern results revealed alternation in the intensity of the protein band of treated and control groups. This difference in intensity is well seen in the band of 25 kDa. In addition, a new band was observed in the protein pattern (between the range of 63–75 kDa).

### 3.4 Determination of protein pattern under environmental conditions

**Fig. 4:**
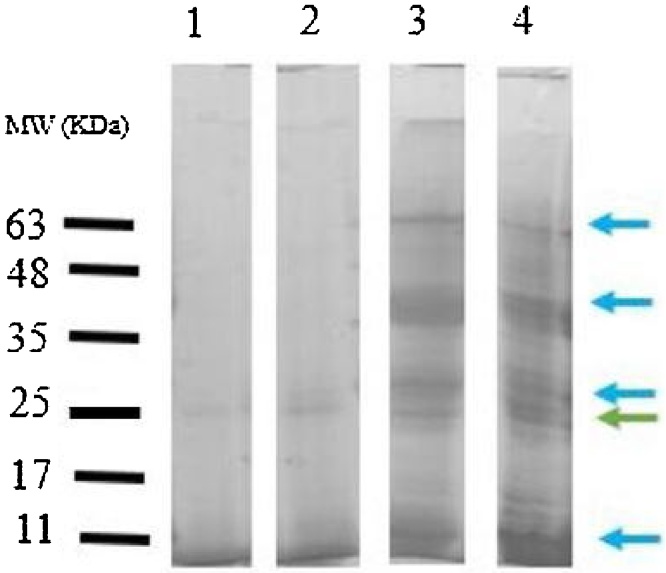
Evaluation of skin mucus protein profile using SDS-PAGE; The green arrow indicates band with enhanced intensity; the blue arrows indicate a new band; Line 1: station 1, Line 2: station 2, Line 3: station 3 and Line 4: station 4.

**Fig. 5:**
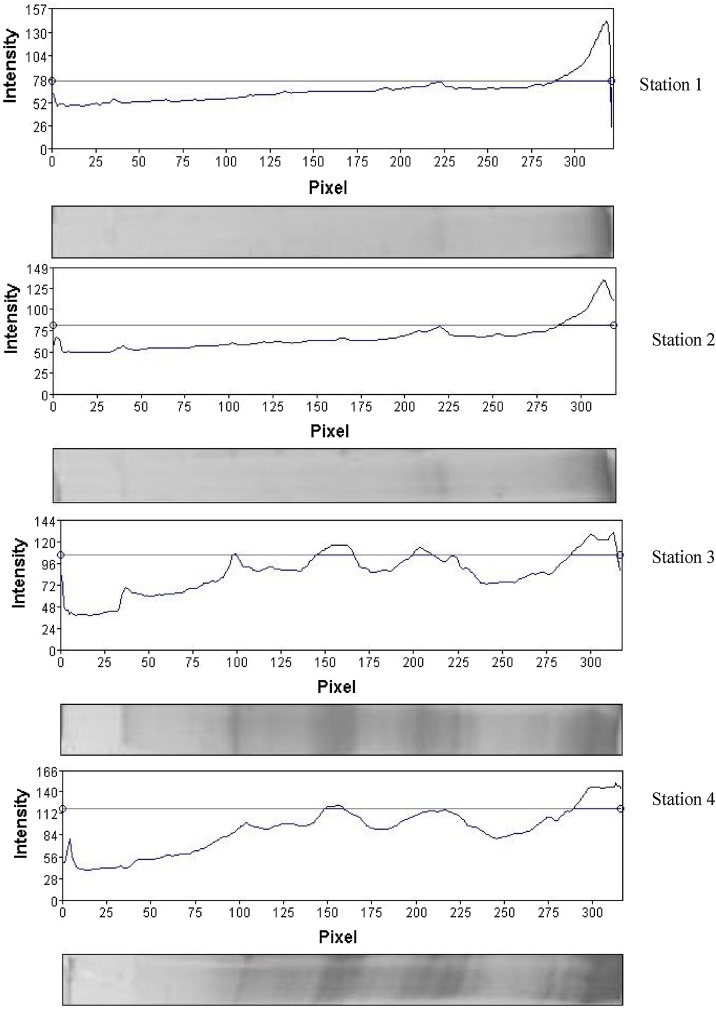
The pattern of skin mucus protein bands at sampling stations in the Gulf of Gorgan.

The SDS-page of the protein pattern of the mucus in Round Goby is displayed in Fig. 4, Fig. 5. Obvious differences were observed between the protein pattern of the contaminated and control stations. Protein pattern results indicated alternation in the intensity of the protein band of the sampling stations. This difference in intensity of band is clearly seen in the band of 25 kDa. New bands were also observed at the contaminated stations, while they were not observed at the control station (within 11–17 and 30−63 kDa).

### 3.5 Measurement of mucus immune parameters at sub-lethal concentrations of lead

Table 3 reports the results of measuring the mucus immune parameters of Round Goby, exposed to the sub-lethal concentrations of lead. Elevation of lead levels under the laboratory conditions led to significant changes in the levels of mucus immune parameters compared to the control group (P < 0.05), with only alkaline phosphatase levels being an exception (P > 0.05). Upon the rise of lead concentration, protein and lysozyme levels showed an increasing trend compared to the control group. On the other hand, with the elevation of lead concentration, first an increasing trend was observed in the level of mucus immunoglobulin M, followed by a decreasing trend, though its decreasing trend was not significant compared to the control group.

**Table 3:** Skin mucus immune parameters of *Neogobius melanostomus* exposed to different concentrations of lead for 14 days.

Parameters	Concentrations			
	Control	T1	T2	T3
	0 mg/ l	3.75mg/ l	7.5mg/l	15mg/l
Protein content (mg/ml)	0.197 ± 0.001^b^	0.217 ± 0.006^a^	0.226 ± 0.018^a^	0.228 ± 0.007^a^
Lysozyme (U/ml)	24.85 ± 2.15^b^	25.50 ± 3.50^b^	34.55 ± 1.45^a^	34.40 ± 1.10^a^
Alkaline phosphatase (U/l)	31.70 ± 0.46^a^	28.77 ± 4.87^a^	31.80 ± 11.55^a^	42.85 ± 0.58^a^
Immunoglobulin M (mg/dl)	2.95 ± 0.20^b^	3.00 ± 0.04^b^	3.24 ± 0.18^a^	2.73 ± 0.07^b^

Different letters indicate significant differences among concentrations ; ANOVA significance: p < 0.05.

The correlation test for the liver tissue lead concentrations and the safety indicators of the skin mucus revealed no significant correlation between the other indicators of mucus immunity and liver lead concentrations, where only lysozyme had a significant and positive correlation (P < 0.05) (Table 4).

**Table 4:** Correlation of skin mucus parameters of *Neogobius melanostomus* with the liver lead during laboratory conditions of exposure to lead.

Parameters	Protein content	Lysozyme	Immunoglobulin M
*Pearson correlation (r)*	0.53	0.7**	−0.45
*Sig(p)*	0.08	0.01	0.14

*Correlation is significant at the 0.05 level, ** Correlation is significant at the 0.01 level.

### 3.6 Measurement of mucus immune parameters under environmental conditions

The levels of protein and immunoglobulin M showed no significant difference in environmental conditions (P > 0.05), while lysozyme and alkaline phosphatase levels of skin mucus showed a significant difference under such conditions (P < 0.05). In this regard, lysozyme levels revealed an increasing trend while alkaline phosphatase levels showed a decreasing trend compared to the control upon contamination intensification (Table 5).

**Table 5:** Skin mucus parameters of *Neogobius melanostomus* obtained from the sampling stations in the Gulf of Gorgan.

Parameters	Stations			
	Control			
	Station1	Station 2	Station 3	Station 4
Protein content (mg/ml)	1.21 ± 0.004^a^	1.11 ± 0.523^a^	1.06 ± 0.030^a^	1.08 ± 0.025^a^
Lysozyme (U/ml)	8.00 ± 1.40^c^	10.65 ± 1.75^bc^	13.63 ± 2.23^b^	16.86 ± 0.85^a^
Alkaline phosphatase (U/l)	190 .73 ± 14.01^a^	182.92 ± 9.60^a^	142.72 ± 2.28^b^	121.98 ± 2.80^c^
Immunoglobulin M (mg/dl)	15.13 ± 0.11^a^	14.73 ± 0.11^a^	15.03 ± 0.36^a^	15.15 ± 0.05^a^

Different letters indicate significant differences among concentrations; ANOVA significance: p < 0.05.

According to the results of the correlation between lead concentrations of liver tissue and mucus immune parameters of Round Goby in environmental conditions, lysozyme levels had a positive correlation while alkaline phosphatase levels had a negative correlation with lead contamination of Round Goby liver under environmental conditions (Table 6).

**Table 6:** Correlation of skin mucus parameters of *Neogobius melanostomus* with the liver lead during environmental conditions of exposure to lead.

Parameters	Lysozyme	Alkaline phosphatase
*Pearson correlation (r)*	0.73**	−0.87**
*Sig(p*	0.007	0.000

*Correlation is significant at the 0.05 level, ** Correlation is significant at the 0.01 level.

The response of mucus immune parameters to lead contamination under both laboratory and environmental conditions was compared. It was found that only lysozyme had the same and significant correlation in both laboratory and environmental conditions, and lysozyme can be introduced as an appropriate and efficient biomarker for mucus immune indicators.

## 4 Discussion

The correlation coefficient between the concentration of lead in the sediment and the concentration of lead in fish liver showed a high numerical value, while the correlation coefficient between the concentration of lead in the water and the concentration of lead in fish liver was lower than the correlation coefficient of sediment. The hydrological study of the Gulf of Gorgan in past research showed that the highest flow rate is related to the inlet mouth of the gulf (more than 0.2 m/s) and in turn the water level fluctuation in the inlet mouth of the gulf. Specifically, the minimum time required for water renewal (less than 1.5 days) occurs at the area near the inlet mouth of the gulf while in other parts of the gulf, this index rises [26].

Meanwhile, lead may precipitate as lead chloride [27]. Note that the fish can absorb metals directly from the water [7,8,28], while only a small portion of free lead ions stay dissolved in the water column. These findings suggest that the study of the concentration of lead in sediments can be a more suitable indicator for bio-accumulation of pollutants in the body of Round Goby. In this study, the level of fish liver lead, which was found under both environmental and laboratory conditions, was used to determine the correlation with the physiological parameters of Round Goby (mucosal indicators), in both environmental and laboratory conditions and eventually to determine the mucosal biomarkers for lead contamination in the Gulf of Gorgan. In general, it was due to the high correlation between lead concentration in liver samples and lead concentration of water under laboratory conditions (r = 0.94, p < 0.01), as well as high correlation between lead concentration in liver samples and lead concentrations in sediments under environmental conditions (r = 0.9, p < 0.01), alongside the water flow (especially at the inlet mouth of the gulf), instability of lead in water and the probability of lead settlement in water under environmental conditions.

### 4.1 Protein pattern

SDS-Page has been widely used in many studies to identify the difference in the protein pattern of many species [[29], [30], [31], [32], [33]]. In this study, the isolation and identification of the molecular weight of different peptides in mucus extracts of Round Goby exposed to different concentrations of lead in both laboratory and environmental conditions were performed by SDS-polyacrylamide gel electrophoresis (SDS-Page). Using SDS-Page, different protein bands were detected in the mucus of Round Goby. Many of these bands were observed to create a new bond in samples with higher levels of contamination than in the control group, especially in the mucus protein pattern of Round Goby under environmental conditions (within 11–17 and 30−63 kDa). Comparison of the results of the pattern of the mucus protein in Round Goby showed that the enhancement of band intensity at 25 kDa was observed under both laboratory and environmental conditions in the protein profile of the mucus. Bands 25−28 kDa can be associated with proteases [16,31]. In general, the difference in the protein pattern of a living creature can be attributed to the internal and external conditions, the types of tissues, and the various stages of evolution.

Guardiola et al. [16] reported a change in the protein pattern of *sparus aurata* skin mucus under the influence of heavy metals, using SDS- Page. It was consistent with the results of the present study concerning the effect of lead contamination on the protein pattern of Round Goby mucus.

### 4.2 Mucus innate immune parameters

The total protein in the samples is indicative of the extent of secretion or mucus levels [34]. Mucus is largely formed in goblet cells [16]. Typically, when mucosal secretions are complete and ready to be released, the nucleus and cellular organs are compressed to the base of the cell. Then, the membranes of these cells, after reaching the epidermis, break from the vertex point and the contents of the cell would be released to the surface of the fish skin [35].

In the present study, under the influence of different concentrations of lead in laboratory conditions, an increase was observed in the amount of mucus protein in Round Goby as compared to the control group, while protein levels of mucus showed no significant difference under environmental conditions. Environmental changes such as exposure to ultraviolet radiation, sudden changes in temperature, pollutants, and acidic waters can also affect the number of goblet cells in the epidermis [36]. Many reports have been published on mucus protein levels in fish [31,37,38]. Accordingly, in the present study, the increasing skin mucus protein under laboratory conditions can be attributed to the rise in the frequency of goblet cells and an increase in the rate of differentiation from lower epidermal cells.

The immunoglobulin M antibody plays an important role in protecting fish against infections [39,40]. Disease outbreaks in fish is increased in polluted waters. Therefore, they react by producing antibodies against bacterial pathogens [41]. In the present study, under laboratory conditions, an increasing trend was observed in the level of immunoglobulin M in Round Goby mucus at different concentrations of lead after which a decreasing trend occurred at high concentrations of contamination, though its decreasing trend was not significant compared to the control group. Similar to the present study, Guardiola et al. [16] observed a significant increase in the immunoglobulin M of *Sparus aurata* mucus exposed to various concentrations of heavy metals. In the present study, there was no significant difference between Immunoglobulin M levels under environmental conditions, which was consistent with the results of Sanchez-Dardon et al. [42]. They investigated the impact of heavy metals of mercury and cadmium on the levels of rainbow trout mucus immunoglobulin for 30 days. In general, quantitative studies have examined the levels of mucus immunoglobulin in fish exposed to contaminated water. Therefore, further research is required to explore the importance of secretion of mucus immunoglobulin M levels and their relationship with contaminated waters.

In the mucus layer, there are many mucus enzymes, which may play a significant role [43,44]. Alkaline phosphatase acts as an antibacterial factor in mucus given its hydrolytic activity, which increases in the mucus of fish after chemical and physical stresses, skin regeneration, along with bacterial and parasitic infections [45]. In the present study, there was no significant difference in the levels of alkaline phosphatase enzyme under laboratory conditions. However, an increase in the amount of lysozyme enzyme was observed in comparison to the control group under both laboratory and environmental conditions, which was consistent with the results of Guardiola et al. [16]. However, it was incongruent with the results of Stabili and Pagelira [46]. In total, the elevation of these enzymes in the skin mucus could contribute to the accumulation of rodlet cells along the skin epithelium, due to the stress status caused by exposure to heavy metals [16].

In many cases, the combined effects of several chemicals in the environment will be different from their individual effects, which can be either synergism or antagonism [47]. In addition, physical and chemical factors affect the toxicity of metals [48]. As such, in the present study, the differences in the results of biochemical parameters between laboratory and environmental conditions, as well as differences in the results of biochemical parameters between different stations, unlike the level of lead at the stations, may be due to the reasons mentioned above.

Overall, the results showed obvious differences between the protein patterns of treatment groups and contaminated stations with the control group under both environmental and laboratory conditions. Among innate immune parameters of the mucus, only lysozyme showed a significant correlation with the concentrations of lead accumulated in the liver of Round Goby under laboratory conditions. It can be a biomarker candidate of mucus immune parameters. Under environmental conditions, lysozyme showed a positive correlation while alkaline phosphatase revealed a negative correlation with lead contamination in the liver of Round Goby. Eventually, in the pattern of mucus protein, the intensity of band at 25 kDa increased in both conditions, suggesting the presence of proteases in this region. Also, among the immune parameters, lysozyme enzyme with a significant and positive correlation with liver lead contamination in both conditions, can be considered as a biomarker of protein profile and safety in Round Goby mucus, for measuring lead contamination in the Gulf of Gorgan.

## Declaration of interests

The authors declare that they have no known competing financial interests or personal relationships that could have appeared to influence the work reported in this paper.

## CRediT authorship contribution statement

**Fakhriyeh Omidi:** Investigation, Writing - review & editing. **Hojatollah Jafaryan:** Supervision. **Rahman Patimar:** Formal analysis. **Mohammad Harsij:** Project administration. **Hamed Paknejad:** Conceptualization, Methodology, Resources.
